# Supplementary material for: Structured information extraction from scientific text with large language models
Source: Nat Commun. 2024 Feb 15;15:1418. doi: 10.1038/s41467-024-45563-x (PMC10869356; doi:10.1038/s41467-024-45563-x)
Supplement: Supplementary file 3 — Reporting Summary [file 41467_2024_45563_MOESM3_ESM.pdf]

nature portfolio

Corresponding author(s): Alexander Dunn (ardunn@lbl.gov)
John Dagdelen
Anubhav Jain (ajain@lbl.gov)

Last updated by author(s): Dec 22, 2023

Reporting Summary

Nature Portfolio wishes to improve the reproducibility of the work that we publish. This form provides structure for consistency and transparency in reporting. For further information on Nature Portfolio policies, see our Editorial Policies and the Editorial Policy Checklist.

Statistics

For all statistical analyses, confirm that the following items are present in the figure legend, table legend, main text, or Methods section.

n/a	Confirmed	
☐	☒	The exact sample size (*n*) for each experimental group/condition, given as a discrete number and unit of measurement
☒	☐	A statement on whether measurements were taken from distinct samples or whether the same sample was measured repeatedly
☒	☐	The statistical test(s) used AND whether they are one- or two-sided<br>*Only common tests should be described solely by name; describe more complex techniques in the Methods section.*
☒	☐	A description of all covariates tested
☐	☒	A description of any assumptions or corrections, such as tests of normality and adjustment for multiple comparisons
☐	☒	A full description of the statistical parameters including central tendency (e.g. means) or other basic estimates (e.g. regression coefficient) AND variation (e.g. standard deviation) or associated estimates of uncertainty (e.g. confidence intervals)
☒	☐	For null hypothesis testing, the test statistic (e.g. *F*, *t*, *r*) with confidence intervals, effect sizes, degrees of freedom and *P* value noted<br>*Give P values as exact values whenever suitable.*
☒	☐	For Bayesian analysis, information on the choice of priors and Markov chain Monte Carlo settings
☒	☐	For hierarchical and complex designs, identification of the appropriate level for tests and full reporting of outcomes
☒	☐	Estimates of effect sizes (e.g. Cohen's *d*, Pearson's *r*), indicating how they were calculated

Our web collection on statistics for biologists contains articles on many of the points above.

Software and code

Policy information about availability of computer code

Data collection	Scopus API via pybliometrics v3.5.2 with Python v3.7.3 was used to scrape publicly available data. MongoDB Server v4.4.18 was used to organize and hold data
Data analysis	We provide all code for data analysis open source at https://github.com/lbnlp/NERRE under an MIT license. We also provide the llama-2 weights at https://figshare.com/articles/dataset/LoRA_weights_for_Llama-2_NERRE/24501331 and the forked code for fine-tuning llama-2 (along with instructions) at https://github.com/lbnlp/nerre-llama. We also provide DOIs for all three of these: https://doi.org/10.5281/zenodo.10421174, https://doi.org/10.5281/zenodo.10421187, https://doi.org/10.6084/m9.figshare. 24501331.v1.

For manuscripts utilizing custom algorithms or software that are central to the research but not yet described in published literature, software must be made available to editors and reviewers. We strongly encourage code deposition in a community repository (e.g. GitHub). See the Nature Portfolio guidelines for submitting code & software for further information.

Data

Policy information about [availability of data](#)

All manuscripts must include a [data availability statement](#). This statement should provide the following information, where applicable:

- Accession codes, unique identifiers, or web links for publicly available datasets
- A description of any restrictions on data availability
- For clinical datasets or third party data, please ensure that the statement adheres to our [policy](#)

All data used for this study are available at https://github.com/LBNLP/NERRE, which contains the annotated datasets, test and train splits, evaluation code, and Jupyter notebooks and python scripts for annotation. Intermediate files for each step of the pipeline reported in this method are stored in this repository with corresponding documentation.

Research involving human participants, their data, or biological material

Policy information about studies with [human participants or human data](#). See also policy information about [sex, gender (identity/presentation), and sexual orientation](#) and [race, ethnicity and racism](#).

Reporting on sex and gender	*Use the terms sex (biological attribute) and gender (shaped by social and cultural circumstances) carefully in order to avoid confusing both terms. Indicate if findings apply to only one sex or gender; describe whether sex and gender were considered in study design; whether sex and/or gender was determined based on self-reporting or assigned and methods used. Provide in the source data disaggregated sex and gender data, where this information has been collected, and if consent has been obtained for sharing of individual-level data; provide overall numbers in this Reporting Summary. Please state if this information has not been collected. Report sex- and gender-based analyses where performed, justify reasons for lack of sex- and gender-based analysis.*
Reporting on race, ethnicity, or other socially relevant groupings	*Please specify the socially constructed or socially relevant categorization variable(s) used in your manuscript and explain why they were used. Please note that such variables should not be used as proxies for other socially constructed/relevant variables (for example, race or ethnicity should not be used as a proxy for socioeconomic status). Provide clear definitions of the relevant terms used, how they were provided (by the participants/respondents, the researchers, or third parties), and the method(s) used to classify people into the different categories (e.g. self-report, census or administrative data, social media data, etc.) Please provide details about how you controlled for confounding variables in your analyses.*
Population characteristics	*Describe the covariate-relevant population characteristics of the human research participants (e.g. age, genotypic information, past and current diagnosis and treatment categories). If you filled out the behavioural & social sciences study design questions and have nothing to add here, write "See above."*
Recruitment	*Describe how participants were recruited. Outline any potential self-selection bias or other biases that may be present and how these are likely to impact results.*
Ethics oversight	*Identify the organization(s) that approved the study protocol.*

Note that full information on the approval of the study protocol must also be provided in the manuscript.

Field-specific reporting

Please select the one below that is the best fit for your research. If you are not sure, read the appropriate sections before making your selection.

☐ Life sciences ☐ Behavioural & social sciences ☒ Ecological, evolutionary & environmental sciences

For a reference copy of the document with all sections, see [nature.com/documents/nr-reporting-summary-flat.pdf](#)

Ecological, evolutionary & environmental sciences study design

All studies must disclose on these points even when the disclosure is negative.

Study description	Note our study is not an ecological, evolutionary, or environmental science study. We examine large language models (LLMs) for extracting complex relational scientific information from raw unstructured materials science text. We describe a new methodology for fine-tuning LLMs to extract this information and demonstrate its use on three example materials science use cases (general materials entities, metal organic frameworks, and inorganic solid-state doping).
Research sample	Our training samples are publicly available scientific abstracts annotated by domain experts (the authors). The doping task's training set contains 413 sentences from 162 abstracts. The metal organic framework task training set is 507 abstracts and the general materials task is 634 abstracts.
Sampling strategy	Train/test splitting was performed by random split.

Data collection	Alexander Dunn and John Dagdelen collected the data by filtering entries from a previously scraped database of scientific abstracts using basic keyword searches as described in the manuscript.
Timing and spatial scale	Data was collected between March 2022 and January 2023, though this does not reflect the publication dates of the scraped abstracts. These scraped abstracts are text data originally published between 1960 and 2022.
Data exclusions	Data for the Doping task were excluded from inference and evaluation if they were deemed irrelevant as per regular expressions .
Reproducibility	We perform cross validation to ensure the generalization performance and reproducibility of the method. Our code and data is publicly available for use at https://github.com/lbnlp/NERRE. The weights for the LLama-2 models are downloadable via https://github.com/lbnlp/nerre-llama including direct links.
Randomization	Train/test splitting was performed by random split.
Blinding	Blinding was not relevant to this study.

Did the study involve field work? ☐ Yes ☒ No

Reporting for specific materials, systems and methods

We require information from authors about some types of materials, experimental systems and methods used in many studies. Here, indicate whether each material, system or method listed is relevant to your study. If you are not sure if a list item applies to your research, read the appropriate section before selecting a response.

Materials & experimental systems

n/a	Involved in the study
☒	☐ Antibodies
☒	☐ Eukaryotic cell lines
☒	☐ Palaeontology and archaeology
☒	☐ Animals and other organisms
☒	☐ Clinical data
☒	☐ Dual use research of concern
☒	☐ Plants

Methods

n/a	Involved in the study
☒	☐ ChIP-seq
☒	☐ Flow cytometry
☒	☐ MRI-based neuroimaging
